# Identification of common and specific cold resistance pathways from cold tolerant and non-cold tolerant mango varieties

**DOI:** 10.7717/peerj.18431

**Published:** 2024-10-30

**Authors:** Jian-hua Wang, Xiaoyan Feng, Muhammad Aleem Ashraf, Yufeng Li, Yu Kong, Qiuliang Cai, Shuli Xian, Huixiang Yin, Nai-tong Yu

**Affiliations:** 1 Guangxi Key Laboratory of Biology for Mango, College of Agriculture and Food Engineering, Baise University, Baise, China; 2 Institute of Tropical Bioscience and Biotechnology, Chinese Academy of Tropical Agricultural Sciences, Haikou, China; 3 Department of Biosciences and Technology, Emerson University, Multan, Pakistan

**Keywords:** Mango, Abiotic stress, Cold resistance, Omics, Ethylene-responsive transcription factor

## Abstract

Mango has frequently encountered severe climate and environmental challenges such as low temperatures, seriously affecting the sustainable development of the industry. In the study, physiological measurements showed that the activities of superoxide dismutase (SOD) and peroxidase (POD) were found to be higher in Jinhuang (JH) mango plants than those of Tainong (TN) mango plants under cold stress, indicating cold tolerant (JH) and non-cold tolerant (TN) mango varieties were firstly determined. Subsequently, transcriptomics showed 8,337 and 7,996 differentially expressed genes (DEGs) were respectively identified in JH and TN mango varieties treated at 4 °C for 36 h, while more DEGs (10,683 and 10,723) were screened when treated at 4 °C for 72 h. Quantitative real-time PCR (qRT-PCR) of the selected DEGs confirmed their transcriptional levels displayed agreement to the transcriptome data. Gene Ontology (GO) and Kyoto Encyclopedia of Genes and Genomes (KEGG) enrichment analyses showed two primary cold resistant regulation pathways, photosynthesis-antenna proteins pathway and photosynthesis pathway, were both significant annotated in the two mango varieties, indicating share the common regulation mechanism response to cold stress. However, five specific cold resistant pathways, such as amino acid and carbohydrate metabolisms, were identified in JH mango variety with cold stress for longer duration, indicating the specific regulation pathways in the cold tolerant mango varieties. Furthermore, 43 ethylene-responsive transcription factors (ERFs) were significantly annotated in JH mango after cold-treated for 72 h comparing with the control group, and three of them ERF109-1, ERF017-1 and ERF017-2 were highly expressed, which may play important regulatory roles in plant cold resistance. These results provided insights into the primary and specific molecular mechanisms of different mango varieties resistance to chill.

## Introduction

Mango (*Mangifera indica* L.) is a tropical fruit with rich nutritional value, such as vitamin C, carotene, dietary fiber, *etc*. Its pulp is delicate and juicy, and releases a unique aroma and sweetness, so it is loved by people around the world (Lebaka et al., 2021). After decades of development, China has now become one of the world’s major mango producing countries. It is widely planted in tropical and subtropical regions of China, including Hainan, Guangdong, Guangxi, Yunnan and Sichuan (https://www.fao.org/faostat/en/#data/QCL).

In nature, plants often suffer from biotic stress of diseases, pests and weeds, and abiotic stress such as high temperature, low temperature, drought and salinity, which seriously affect the yield and quality of fruits (Dofuor et al., 2023; Mulungu et al., 2023; Kan et al., 2023; Islam et al., 2022). Temperature is the main decisive factor affecting plant growth and regional distribution of plants (Zhang et al., 2023a), and it is of great significance to study its impact on tropical agricultural production and food security. Mango is a tropical evergreen fruit tree, which prefers high temperature 25~35 °C, and the optimum growth temperature for mango is among 24~27 °C. The plants and their fruits will be damaged by cold stress when the temperature is lower than 4 °C, especially young trees with high sensitivity to the low temperature (Liu et al., 2023). Furthermore, the planting area of mango in China mainly located in the northern margin of the earth. In winter, it is periodically threatened by natural disasters such as cold damage or frost, which has become an important factor affecting mango production in China (Kong et al., 2024). In recent years, mango has frequently encountered severe climatic and environmental challenges, which cause huge economic losses to farmers or agricultural enterprises and seriously affect the sustainable development of the industry (Wei et al., 2017; Abdel-Aziz et al., 2023).

The damage caused by low temperature to plants can be divided into cold damage and freezing damage. Cold damage affects plant photosynthesis, inhibits intracellular enzyme activity, imbalances of reactive oxygen species and endogenous hormones, and even causes partial cell death in severe cases. When the temperature is below 0 °C, it can cause freezing inside the cell, and ice crystals can puncture the cell membrane, resulting in cell dehydration, cell death and tissue necrosis, and even plant death (Hincha & Zuther, 2020; Takahashi, Uemura & Kawamura, 2018). Mango fruit trees are perennial plants. Therefore, low temperature will not only cause death of flowers, buds, leaves and branches in the first year, but also weaken the tree, leading to plant death, *etc*., affecting the growth of the plants in the following years. Studies have shown that the damage of low temperature to fruit trees mainly destroys the structure of cell membrane, thus reducing its fluidity and leading to the inactivation of ATPase. At the same time, it also causes membrane lipid phase transition, changes the arrangement of membrane proteins and membrane lipids, and increases the permeability of the cells. Eventually, the physiological and biochemical processes of the plant cell membrane structure are continuously disrupted, resulting in osmotic-pressure anomaly, cells dehydration, plant death (Kidokoro, Shinozaki & Yamaguchi-Shinozaki, 2022).

However, plants can adapt to low temperature and cold environment through genetic variation and natural selection for a long period of time, thus evolving cold resistance pathways (Sasaki & Imai, 2023). At present, high-throughput omics analysis such as genomics, transcriptomics, proteomics and metabolomics have been widely combined to study various abiotic stresses in plants and deepen the understanding of the different biological regulation pathways. Transcriptome high-throughput sequencing, as a modern genetic research tool, is widely used to analyze the mRNA expression of organism or individual in specific biological processes and reveal their internal molecular mechanisms. So far, many studies have been carried out on the transcriptional response of plants to low temperature stress, including pineapple (Hong et al., 2023), banana (Zhu et al., 2023) and other tropical crops. These studies have found a large number of cold stress-related genes involved in adapting to low temperature environment. Some scholars have reported that molecular process responses to low temperature stress in many plants, including gene expression, redox state and complex signal transduction, such as ERF involving in the regulation of biotic and abiotic stresses (Gan et al., 2019; Hu et al., 2020).

At present, research progresses of mango plants resistance to cold stress mainly focuses on the stress identification and evaluation of different germplasm resources, measurement of physiological and biochemical indexes changes, cold-resistant genes and pathways mining, cold damage prevention and control measures, *etc.,* (Sudheeran et al., 2018; Yamanaka et al., 2019; Gafni et al., 2022; Zhang et al., 2023b). However, the molecular mechanism of cold resistance in mango plants is still unknown. In this study, to provide a theoretical basis for the genetic improvement of the cold resistance traits and the prevention of chilling damage in mango production, the non-cold resistant Tainong mango and the cold-resistant Jinhuang mango varieties were used as experimental objects, and the significant DEGs response to low temperature stress and the enrichment KEGG pathways were identified to elucidate the molecular regulation mechanism by using transcriptomics and molecular biology methods. This study provides new insights into the adaptation of mango to low temperature, and promotes the breeding of tropical plants to stress-resistant varieties.

## Materials and Methods

### Plant materials and cold stress

Two mango varieties, Jinhuang (JH) and Tainong (TN), were used to analyze the resistance mechanisms against cold stress. A total of 15 seedlings of JH (61.20 cm ± 8.35) and 15 seedlings of TN (57.00 cm ± 6.75) were obtained from Hainan Baizhou Agriculture Co., LTD, Danzhou, China. Each seedling was being added 50 mL of H_2_O and was pre-treatment in a greenhouse at 24 °C for 7 days. Subsequently, the 15 seedlings of JH were devided into three groups on average, namely JH4-1 group, JH4-2 group and JH24 group, while the 15 seedlings of TN were devided into three groups on average as well, namely TN4-1 group, TN4-2 group and TN24 group.

Among these groups, JH4-1, JH4-2, TN4-1, and TN4-2 were placed in a low temperature culture room at 4 °C for 36 h (JH4-1 and TN4-1 groups) or 72 h (JH4-2 and TN4-2 groups) for cold stress treatment. The two control groups of JH24 and TN24 were maintained in the greenhouse at 24 °C for 72 h. The leaves of treated samples were collected at 36 h (JH4-1 and TN4-1 groups) or 72 h (JH4-2 and TN4-2 groups). Control samples were also collected from the JH24 group and TN24 group. Five leaf samples were collected from each group. All the samples were immediately frozen in liquid nitrogen and stored at −80 °C for further use.

### Superoxide dismutase and peroxidase

Before carrying out transcriptome sequencing, the physiological and biochemical indicators of two mango varieties should be determined. In the study, two time points of superoxide dismutase (SOD) and peroxidase (POD) activities of JH mango and TN mango varieties were used to judge this standard under adverse conditions. Each leaf of 30 mango plants was selected after cold stress, and every leaves from the same group were mixed for evaluating the physiological indices in both mango varieties. The activities of SOD (Catalog No. G0101F, Grace, Suzhou, China) and POD (Catalog No. G0107F, Grace, Suzhou, China) were measured using the kits provided by Suzhou Grace Biotechnology Co., Ltd. The statistical significance of SOD or POD value in different groups was determined by Student’s t test. Values of *P* < 0.05 were considered significant.

### RNA preparation and qualification

Total RNAs from mango leaves were extracted by using RNAprep Pure Plant Plus Kit (Polysaccharides & Polyphenolics-rich) according to the manufacturer’s instruction (Catalog No. DP441, Tiangen, Beijing, China), and further treated with DNase I (Catalog No. RT411, Tiangen, Beijing, China) to remove DNA contamination. The RNA concentration and purity were measured using Nanodrop2000 (Thermo Fisher Scientific, MA, USA). The RNA integrity (RIN) was assessed using the Caliper LabChip GX system (Perkin Elmer, Waltham, MA, USA).

### Library preparation for transcriptome sequencing

A total amount of ~3.5 μg RNA per sample was used as input material for the cDNA library construction. Sequencing libraries were generated using Hieff NGS Ultima Dual-mode mRNA Library Prep Kit (Catalog No. 13533ES96, Yeasen, Shanghai, China) as following three steps: firstly, the enrichment, purification, and fragmentation of mRNA were performed; secondly, the first strand cDNA was synthesized for preparing the second strand cDNA, which includes end repair and dA-tailing; thirdly, adaptor primers adpter3 and adpter5 (Table 1) were linked to the two ends of ds cDNA and the products were purified. Finally, the cDNA libraries were further amplified and purified for the library quality inspection by using Qsep400 standard DNA clip kit in Qsep-400 instrument.

**Table 1:** The list of primers in this study.

Primer name	Primer sequence (5′-3′)	Usage	Length	GenBank accession Nos.	Primer nt identity
adpter3	AGATCGGAAGAGCACACGTCTGAACTCCAGTCAC	Adaptor primers for libraries	/	/	/
adpter5	AGATCGGAAGAGCGTCGTGTAGGGAAAGAGTGT			/	/
β-actin-qF	GAATATGAAACTGCCCCTTGC	RT-qPCR	109 bp	XM_044618540.1	100%
β-actin-qR	CTTCCCGAAATAGACCTGATCC			XM_044618540.1	100%
XTH23-qF	CGCCTTCACTCCCACTATAATC	RT-qPCR	177 bp	XM_044608360.1	100%
XTH23-qR	GCCATCTCCCCAAGTGATATC			XM_044608360.1	100%
TS12-qF	AGACACCATCCACAAAGAGC	RT-qPCR	189 bp	XM_044647035.1	100%
TS12-qR	CTTTTCCCTGTTCTCGCATTC			XM_044647035.1	100%
RADIALIS-4-qF	ATTCCTTTCCCCATCTCTTCG	RT-qPCR	136 bp	XM_044632729.1	100%
RADIALIS-4-qR	AAGAAAGGTGGAGTTGTGGAG			XM_044632729.1	100%
BON1-2-qF	CAGTGAGAGTAAATACGCCAGG	RT-qPCR	134 bp	XM_044638010.1	100%
BON1-2-qR	CCATGAACAAGACCCATATCCC			XM_044638010.1	100%

The prepared libraries were sequenced on an Illumina NovaSeq 6000 platform (Illumina, San Diego, CA, USA) using NovaSeq 6000 S4 Reagent Kit (Illumina, San Diego, CA, USA), and 150-bp paired-end reads were generated (Modi et al., 2021).

### Data quality control of the transcriptomic data

Based on sequencing-by-synthesis (SBS) technology, the significant amounts of raw data with high-quality were generated. Raw data was saved in fastq format. Raw reads in the fastq format were firstly cleaned to remove the adaptor sequences and low quality reads (ploy-Ns ratio greater than 10%, or bp value of Q ≤ 10 greater than 50%) for generating the clean data. Q30 and GC content of the clean data were calculated to evaluate the overall quality of the clean reads. All the downstream analyses were based on the clean data with high quality (Liu et al., 2021a).

### Mapping analysis of the transcriptomic sequencings

Reference genome of mango was downloaded for the RNA-Seq mapping analysis. Version information of the reference genome is Mangifera_indica.v4.0.genome.fa (Wang et al., 2020). HISAT2 (Kim et al., 2019), a Burrows-Wheeler Transform and Ferragina-Manzini (FM) index based search, was used to map clean reads in the reference genome to obtain the localization information of reads on the mango genome. StringTie was applied to assemble the mapped reads for subsequent analysis (Pertea et al., 2015). It utilizes a novel network flow algorithm as well as an optional *de novo* assembly step to assemble and quantify transcripts representing multiple spliced variants for each gene locus.

### Identification of differentially expressed genes

Fragments Per Kilobase of transcript per Million fragments mapped (FPKM) was applied to measure the expression level of a gene or a transcript by StringTie using maximum flow algorithm. In the study, the differential expression genes (DEGs) analysis was performed on comparing the JH4-1 group with the JH24 control group and comparing the JH4-2 group with the JH24 group, respectively. Meanwhile, the DEGs analysis was also performed on comparing the TN4-1 group with the TN24 control group and comparing the JH4-1 group with the JH24 group, respectively. The four comparing groups were named JH24 *vs*. JH4-1, JH24 *vs*. JH4-2, TN24 *vs*. TN4-1, TN24 *vs*. TN4-2.

In the study, transcripts that increased or decreased with a fold change (FC) ≥ 2 and false discovery rate (FDR) < 0.01 were considered to be differentially expressed. DESeq2 accepted input of the clean reads and the DEGs between the experimental group and control group were screened (Liu et al., 2021b). The hierarchical clustering map was used to show the distribution of DEGs, and cluster analysis was used to judge the expression pattern of each gene.

### Verification of transcription levels by real-time quantitative reverse transcription PCR

To confirm gene expression differences of four comparing groups (JH24 *vs*. JH4-1, JH24 *vs*. JH4-2, TN24 *vs*. TN4-1, TN24 *vs*. TN4-2) by transcriptomic sequencing, four DEGs of *probable xyloglucan endotransglucosylase/hydrolase protein 23* (*XTH23*), *probable terpene synthase 12* (*TS12*), *BON1-associated protein 2-like* (*BON1-2*), *RADIALIS-like 4 protein* (*RADIALIS-4*) were selected for real-time quantitative reverse transcription PCR (qRT-PCR) analysis using the primers in Table 1. Total RNA was extracted from plant leaves by using a FastPure Universal Plant Total RNA Isolation Kit (Vazyme, Nanjing, China) according to the manufacturer’s instructions. Two μg RNA (360~500 ng/μL) per sample was transcribed into first strand cDNA by using a HiScript III 1st Strand cDNA Synthesis Kit (+gDNA wiper) (Vazyme, Nanjing, China). Five leaf samples of each group were mixed into a mixture, and three technical replicates were performed for each mixed sample. The amplification program was conducted on a StepOne real-time PCR system (Applied Biosystems, Waltham, MA, USA) and the cycle condition was as follows: 95 °C for 5 min, followed by 40 cycles of 95 °C for 15 s, and 60 °C for 1 min. The internal control gene, *β-actin*, was used to normalize each gene. Transcription levels of selected DEGs were evaluated based on the 2^−ΔΔCt^ method. The statistical significance of qRT-PCR was determined by Student’s t test. Values of *P* < 0.05 were considered significant.

### Gene Ontology and Kyoto Encyclopedia of Genes and Genomes enrichment analyses of DEGs

GO enrichment analysis of four comparing groups (JH24 *vs*. JH4-1, JH24 *vs*. JH4-2, TN24 *vs*. TN4-1, TN24 *vs*. TN4-2) was conducted by the topGO to analyze the functional classification of DEGs. GO terms were extracted from the best hits obtained from BLASTx against the non-redundant database using Blast2GO (Conesa et al., 2005). Then the obtained DEGs were sorted by GO categories using in-house Perl scripts. GO terms with a *P* value below 0.05 were considered significantly enriched.

Meanwhile, the KEGG database (http://www.genome.jp/kegg/genome) was used to analyze the molecular pathway of these four comparing groups, and DEGs with a *P* value below 0.05 in the KEGG pathways considered to be significantly enriched using ClusterProfiler software (Wu et al., 2021).

### Phylogenetic tree construction of ERFs

ERF is a class of protein belonging to the AP2/ERF transcription factor superfamily involved in the regulation of biotic and abiotic stresses in plants. In order to explore the up-regulated ERFs (log_2_FC > 2) in cold-tolerant mango varieties, all *ERF*-related genes with differential expression were screened from JH24 *vs*. JH4-2 group. Phylogenetic tree was constructed based on the amino acid sequences to determine the phylogenetic relationship of all ERFs in JH mango under cold stress. The protein informations of 43 ERFs were listed in Table S1. All amino acid sequences were aligned using ClustalX, then passed to MEGA 11 for tree building using maximum likelihood method with 1,000 bootstrap replicates. Phylogenetic analysis was conducted in MEGA11 (Tamura, Stecher & Kumar, 2021).

## Results

### The activities of SOD and POD of mango plants under cold stress

**Figure 1:**
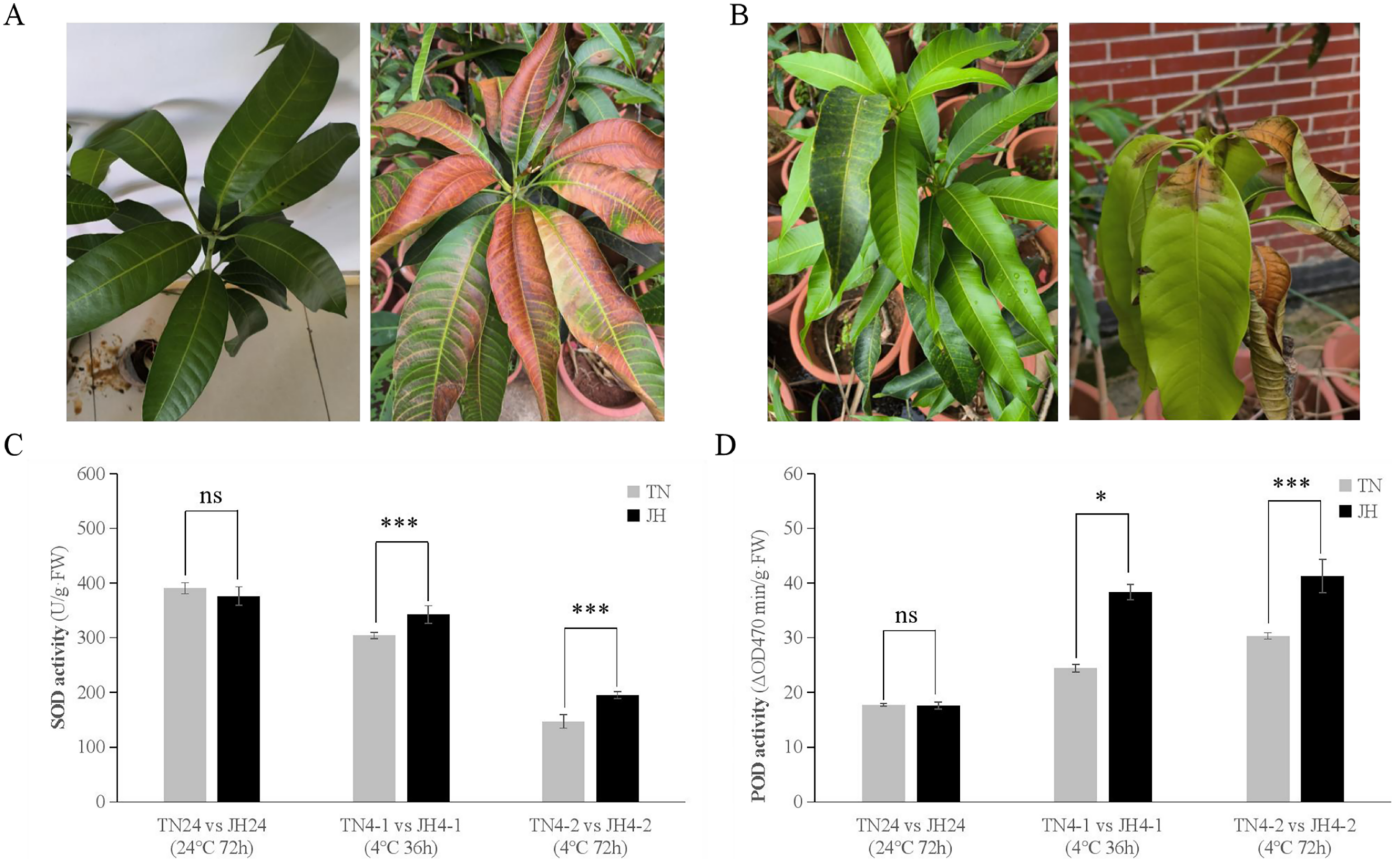
Phenotypic symptoms and physiological responses of JH and TN mango varieties responding to cold stress and untreated group. (A) The phenotypic symptom of JH mango plants displayed asymptomatic damage at 4 °C for 72 h (left), but leaf tips displayed browning after transferred back at room temperature (RT) for 3–5 days (right), (B) The phenotypic symptom of TN mango plants displayed asymptomatic damage at 4 °C for 72 h (left), but leaf bases displayed wither and rot after transferred back at RT for 3–5 days (right), (C) peroxidase (SOD) activity, (D) superoxide dismutase (POD) activity. ***p ≤ 0.01, *p ≤ 0.05, and ns mean non-significant.

The phenotypic observation showed that JH mango plants of both JH4-1 group and JH4-2 group displayed asymptomatic damage, but leaf tips displayed browning after transferred back at room temperature (RT) for 3–5 days (Fig. 1A). Meanwhile, no obvious symptoms were observed on TN mango plants of TN4-1 group or TN4-2 group, but leaf bases displayed wither and rot at 3–5 days after being transferred back at RT (Fig. 1B).

At physiological levels, JH mango plants performed better than TN mango plants responses to the low temperature treatment. For instance, comparing TN4-1 group with JH4-1 group, and comparing TN4-2 group with JH4-2 group, the activities of SOD were found to be higher in JH mango plants than that of TN mango plants under cold stress. However, there was no significant difference in SOD activities between the two mango varieties at 24 °C for 72 h (Fig. 1C). Similar results were observed in the POD activities of the two mango varieties. In detail, the activities of POD were found to be significant differences between the JH mango plants and the TN mango plants at 4 °C treatment for 36 h or 4 °C treatment for 72 h, while no significant difference of the two groups at 24 °C for 72 h (Fig. 1D). These findings showed that JH mango is a more cold-tolerant variety than TN and could mitigate the adverse effects of low temperature by increasing the activities of antioxidant defense enzymes.

### Transcriptomic data quality and mapping analysis

The high-quality of 30 total RNA samples from mango leaves (Conc = 150.60~514.70 ng/μL; OD_260/280_ = 2.01~2.15; RIN = 8.10~8.70) were obtained and conformed to the cDNA library preparation requirement. A large number of raw reads were generated from an Illumina NovaSeq 6000 platform and about 20,000,000 clean reads of each sample were further generated. In total, the GC content was 42.03~43.85% and Q30 percentage was over 93.10% for each sample (Table 2), indicating that high quality transcriptomic sequencing clean data was obtained. The clean data of each sample is available in the GenBase in National Genomics Data Center, Beijing Institute of Genomics, Chinese Academy of Sciences/China National Center for Bioinformation, under accession number CRA016366 that is publicly accessible at https://ngdc.cncb.ac.cn/gsa/browse/CRA016366 (CNCB-NGDC Members & Partners, 2024).

**Table 2:** Summary of the transcriptome data from mango leaves of cold resistant variety (Jinhuang, JH) and non-cold resistant variety (Tainong, TN) with low temperatures treatment.

Group	Samples	Clean reads	Clean bases	GC content	% ≥ Q30	Treatment
JH24	JH24-1	20,690,998	6,191,931,316	43.81%	94.04%	Treatment at 24 °C for 72 h
	JH24-2	22,740,155	6,802,674,662	43.85%	95.87%	
	JH24-3	26,627,548	7,963,214,582	43.56%	94.11%	
	JH24-4	19,360,036	5,794,480,296	43.61%	93.89%	
	JH24-5	20,722,438	6,201,795,334	43.69%	94.05%	
JH4-1	JH4-1-1	20,859,908	6,238,506,022	43.23%	94.04%	Treatment at 4 °C for 36 h
	JH4-1-2	19,727,979	5,900,217,082	42.72%	93.93%	
	JH4-1-3	22,612,265	6,763,548,252	43.39%	96.38%	
	JH4-1-4	20,939,664	6,263,632,400	42.54%	94.33%	
	JH4-1-5	22,126,488	6,618,948,418	43.24%	96.04%	
JH4-2	JH4-2-1	20,794,165	6,221,499,546	42.66%	94.38%	Treatment at 4 °C for 72 h
	JH4-2-2	21,067,749	6,298,223,294	42.03%	95.83%	
	JH4-2-3	20,303,738	6,072,163,702	42.98%	93.91%	
	JH4-2-4	21,356,777	6,376,778,350	43.06%	94.15%	
	JH4-2-5	20,885,262	6,246,790,516	43.37%	94.03%	
TN24	TN24-1	19,179,465	5,741,047,294	43.52%	93.10%	Treatment at 24 °C for 72 h
	TN24-2	19,539,704	5,846,807,346	43.51%	93.37%	
	TN24-3	22,585,139	6,749,521,950	43.29%	93.79%	
	TN24-4	23,735,944	7,095,142,634	43.62%	94.54%	
	TN24-5	23,910,654	7,157,151,728	43.30%	93.75%	
TN4-1	TN4-1-1	20,707,435	6,193,858,750	42.96%	93.79%	Treatment at 4 °C for 36 h
	TN4-1-2	23,302,615	6,950,245,902	43.16%	94.55%	
	TN4-1-3	22,389,472	6,681,149,224	43.51%	94.13%	
	TN4-1-4	20,855,891	6,236,494,380	43.30%	94.45%	
	TN4-1-5	22,463,201	6,717,550,880	43.00%	95.94%	
TN4-2	TN4-2-1	23,419,329	6,998,287,042	42.35%	93.96%	Treatment at 4 °C for 72 h
	TN4-2-2	20,533,795	6,140,930,166	42.10%	93.54%	
	TN4-2-3	19,563,471	5,851,295,024	42.04%	93.28%	
	TN4-2-4	20,426,507	6,104,773,202	42.38%	94.03%	
	TN4-2-5	20,547,675	6,142,268,260	42.10%	93.69%	

### Screening and cluster analysis of the transcriptomic DEGs

**Figure 2:**
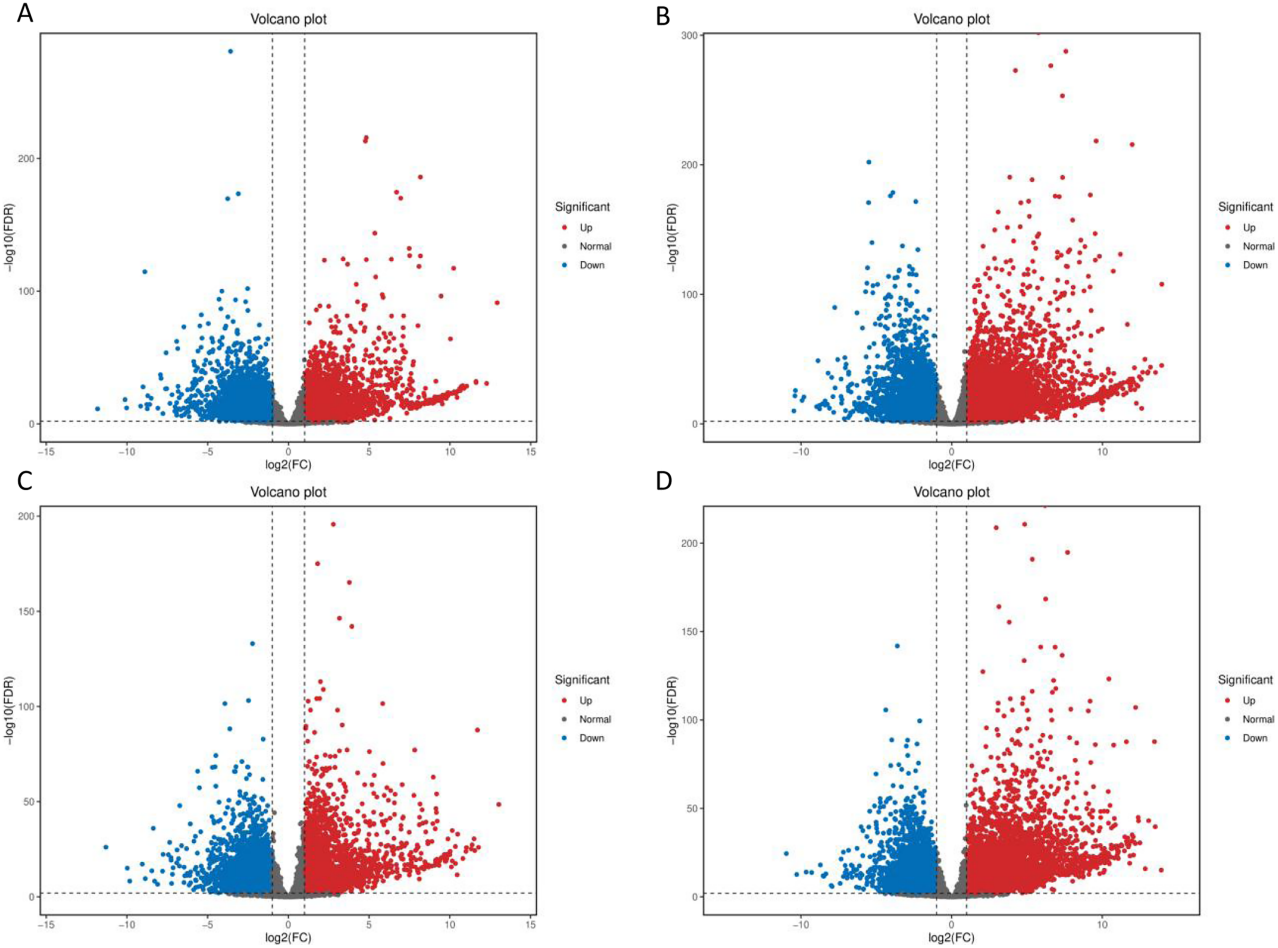
Volcano plot of DEGs between low temperature treatment group and control group in different mango varieties. (A) JH24 group *vs* JH4-1 group, (B) JH24 group *vs* JH4-2 group, (C) TN24 group *vs* TN4-1 group, (D) TN24 group *vs* TN4-2 group. The abscissa log_2_(FC) represents the fold change, while the ordinate −log_10_(FDR) represents the false discovery rate. In the study, log_2_(FC) ≥ 2 and −log_10_(FDR) < 0.01 indicates the differentially expressed genes.

**Figure 3:**
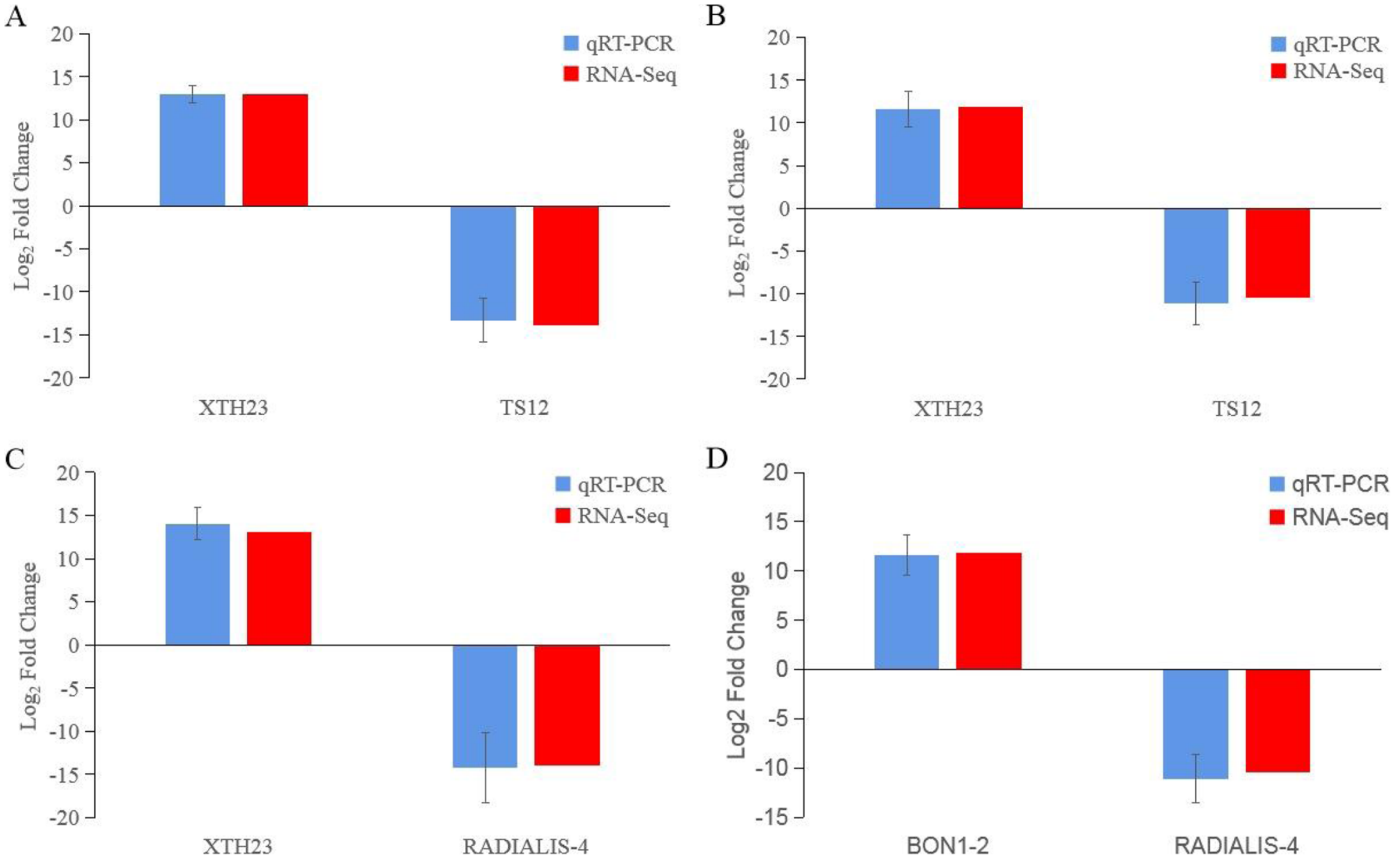
Comparison of the transcription levels of selected DEGs between qRT-PCR (blue) and Illumina NovaSeq 6000 sequencing (red). (A) JH24 group *vs* JH4-1 group, (B) JH24 group *vs* JH4-2 group, (C) TN24 group *vs* TN4-1 group, (D) TN24 group *vs* TN4-2 group. Target gene abbreviations are as follows: *probable xyloglucan endotransglucosylase/hydrolase protein 23 (XTH23), probable terpene synthase 12 (TS12), BON1-associated protein 2-like (BON1-2), RADIALIS-like 4 protein (RADIALIS-4)*. The internal control gene, *β-actin*, was used to normalize each gene. Transcription levels of selected DEGs were evaluated based on the 2^−ΔΔCt^ method. Error bars indicated standard deviations of averages from three replicates. Value above and below the abscissa represent up-regulation and down-regulation, respectively.

After low temperature treatment, a large number of DEGs were identified in both varieties. For the JH mango variety, 8,337 DEGs (4,051 up-regulated and 4,286 down-regulated) were identified from the JH24 *vs*. JH4-1 using DESeq2 software based on FC and FDR values, while 10,683 DEGs (5,552 up-regulated and 5,131 down-regulated) were further identified in JH24 *vs*. JH4-2, indicating more DEGs were screened when treated at 4 °C for longer duration (Fig. 2 and Table 3). Further analysis revealed that both the highest up-regulated genes were *XTH23*, with log_2_FC of 12.93 and 13.93, respectively. The highest down-regulated genes were both of *TS12*, with log_2_FC of −11.82 and −10.47, respectively. The qRT-PCR showed the consistent results (Fig. 3).

**Table 3:** Statistics of DEGs between low temperature (4 °C) treatment group and control (24 °C) group in different mango varieties.

Group	DEG number	NR	GO	KEGG	Up-regulated	Down-regulated
JH24 *vs*. JH4-1	8,337	8,030	6,764	5,748	4,051	4,286
JH24 *vs*. JH4-2	10,683	10,212	8,533	7,258	5,552	5,131
TN24 *vs*. TN4-1	7,996	7,714	6,517	5,595	3,763	4,233
TN24 *vs*. TN4-2	10,723	10,312	8,696	7,406	5,674	5,049

For the TN mango variety, 7,996 DEGs (3,763 up-regulated and 4,233 down-regulated) were identified from TN24 *vs*. TN4-1, while 10,723 DEGs (5,674 up-regulated and 5,049 down-regulated) were further identified in TN24 *vs*. TN4-2, also indicating more DEGs were screened when treated at 4 °C for longer duration (Fig. 2 and Table 3). Further analysis revealed that the highest up-regulated gene was *XTH23* (log_2_FC of 13.04) in TN24 *vs*. TN4-1, while it was *BON1-2* (log_2_FC of 13.91) in TN24 *vs*. TN4-2. The highest down-regulated genes were both of *RADIALIS-4*, with log_2_FC of −11.03 and −10.97, respectively. This outcome was also confirmed by the qRT-PCR (Fig. 3).

### GO and KEGG enrichment analyses of DEGs

**Figure 4:**
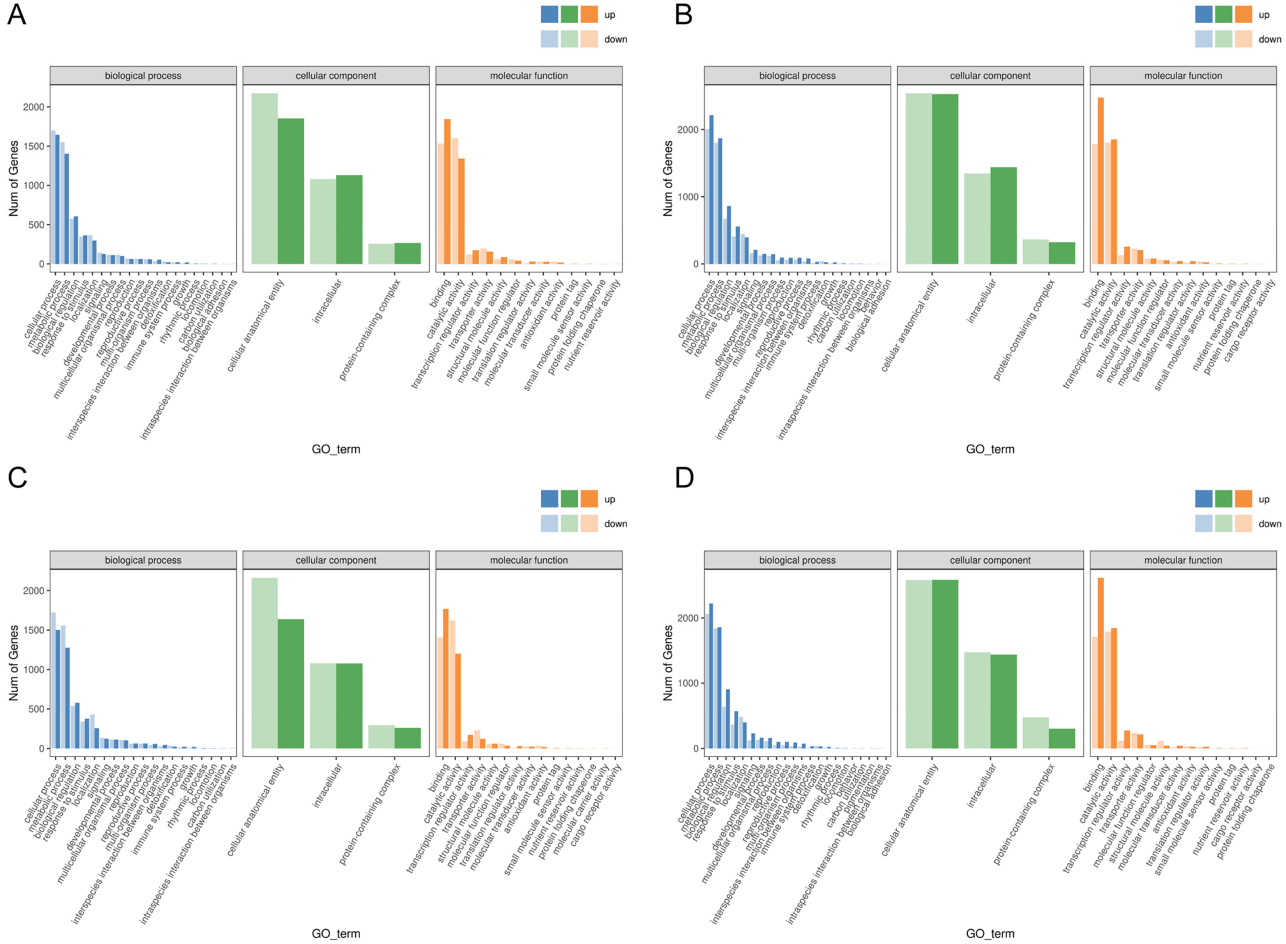
Statistics of GO annotation classification of DEGs between low temperature treatment group and control group in different mango varieties. (A) JH24 group *vs* JH4-1 group, (B) JH24 group *vs* JH4-2 group, (C) TN24 group *vs* TN4-1 group, (D) TN24 group *vs* TN4-2 group.

**Figure 5:**
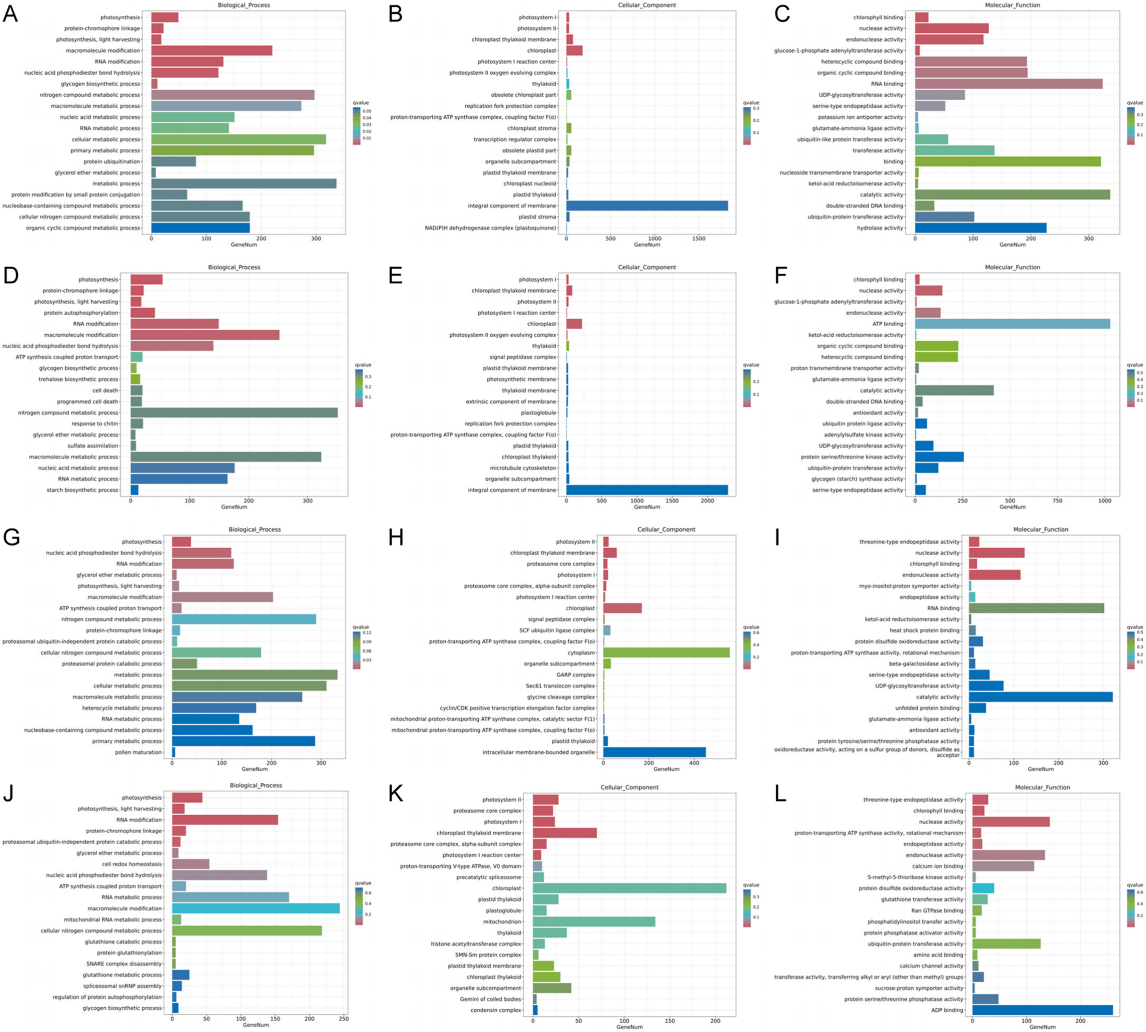
Histogram of GO enrichment annotation of DEGs between low temperature treatment group and control group in different mango varieties. (A) Biological process of JH24 group *vs* JH4-1 group, (B) Cellular component of JH24 group *vs* JH4-1 group, (C) Molecular function of JH24 group *vs* JH4-1 group, (D) Biological process of JH24 group *vs* JH4-2 group, (E) Cellular component of JH24 group *vs* JH4-2 group, (F) Molecular function of JH24 group *vs* JH4-2 group, (G) Biological process of TN24 group *vs* TN4-1 group, (H) Cellular component of TN24 group *vs* TN4-1 group, (I) Molecular function of TN24 group *vs* TN4-1 group, (J) Biological process of TN24 group *vs* TN4-2 group, (K) Cellular component of TN24 group *vs* TN4-2 group, (L) Molecular function of TN24 group *vs* TN4-2 group.

Functional analysis of the DEGs showed that 31 GO terms (q value < 0.05) were annotated in JH24 *vs*. JH4-1, and nine of them associated with photosynthesis or chloroplast. Furthermore, 62.5% (10/16) of the GO terms were annotated into photosynthesis or chloroplast pathways in JH24 *vs*. JH4-2 when JH treated at 4 °C for longer duration. In TN24 *vs*. TN4-1, nine of them (9/46, 19.6%), the same GO terms as JH24 *vs*. JH4-1, were annotated. In addition, eight (8/16, 50.0%) photosynthesis or chloroplast associated GO terms were identified when TN treated at 4 °C for longer duration (Figs. 4 and 5). These results indicate that the most annotated photosynthesis-related or chloroplast-related GO terms respond to the cold stress in mango plants.

**Figure 6:**
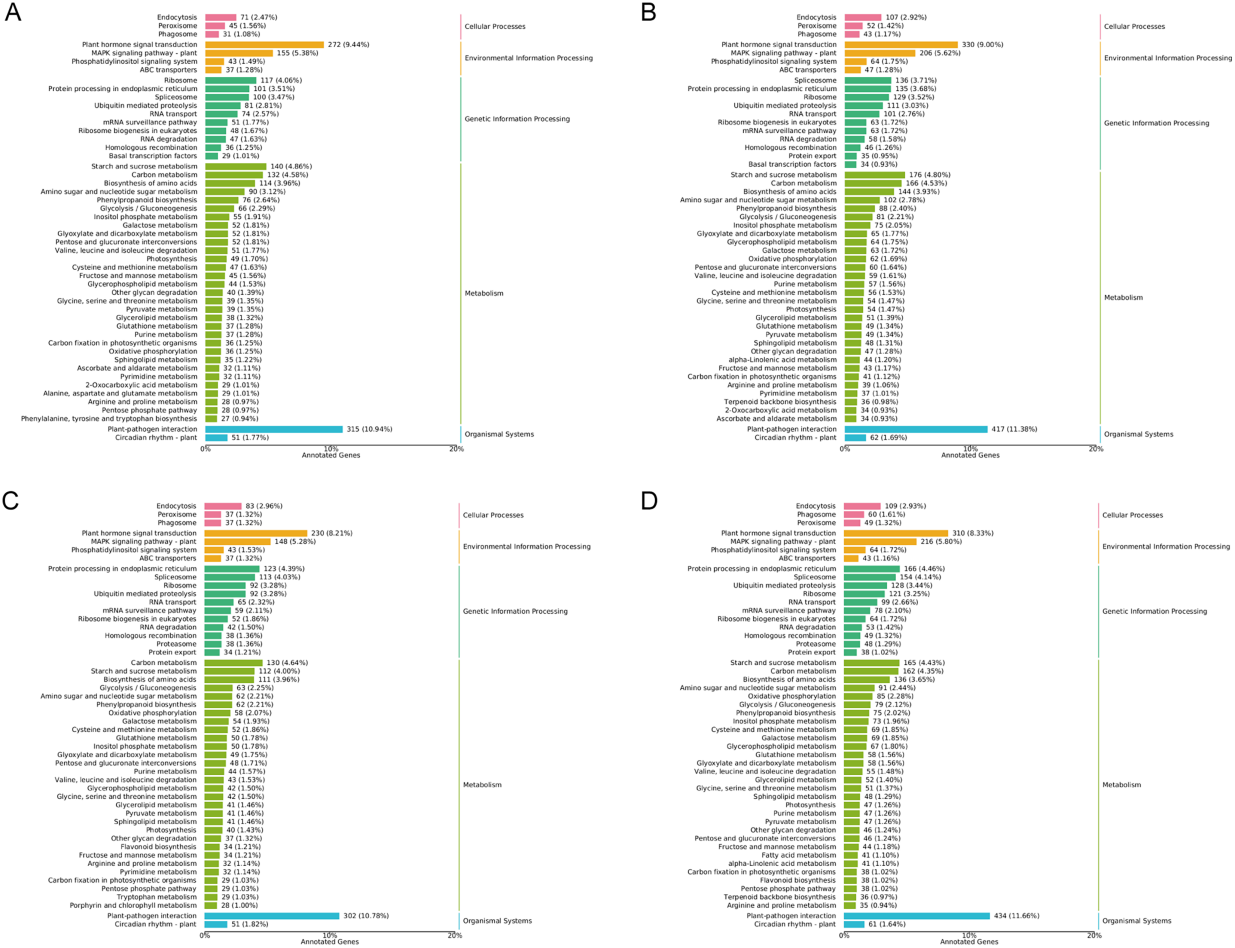
Statistics of KEGG annotation classification of DEGs between low temperature treatment group and control group in different mango varieties. (A) JH24 group *vs* JH4-1 group, (B) JH24 group *vs* JH4-2 group, (C) TN24 group *vs* TN4-1 group, (D) TN24 group *vs* TN4-2 group.

**Figure 7:**
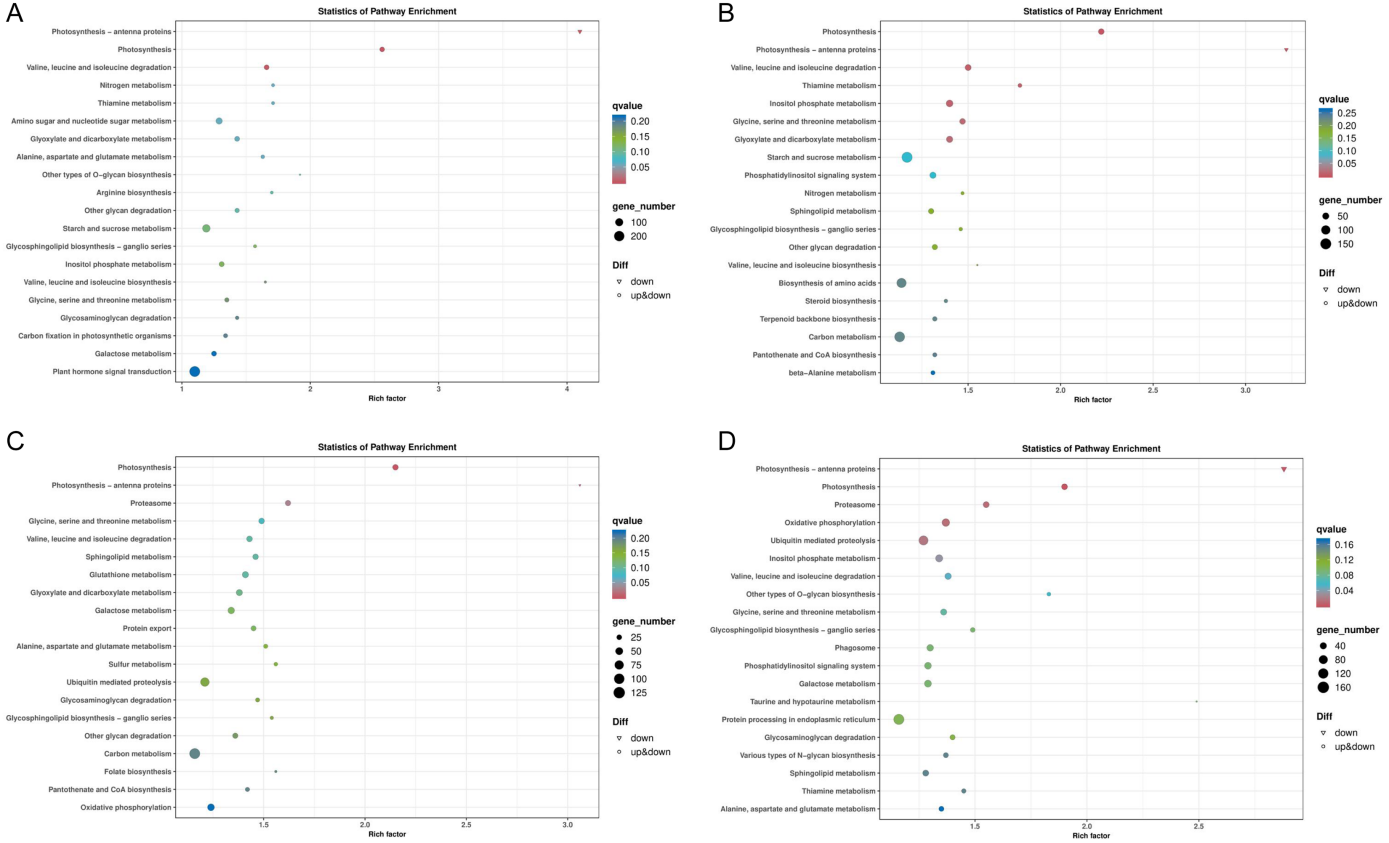
Bubble chart of KEGG enrichment pathways between low temperature treatment group and control group in different mango varieties. (A) JH24 group *vs* JH4-1 group, (B) JH24 group *vs* JH4-2 group, (C) TN24 group *vs* TN4-1 group, (D) TN24 group *vs* TN4-2 group. Note: Each dot represents a KEGG pathway. Y-axis: Pathway; X-axis: Rich factor.

In general, more pathways were enriched and annotated when two varities were treated at low temperature for longer duration. KEGG enrichment annotation analysis was performed on DEGs from different comparison groups of JH24 *vs*. JH4-1, JH24 *vs*. JH4-2, TN24 *vs*. TN4-1, and TN24 *vs*. TN4-2. The results showed that 132, 134, 131 and 133 pathways were annotated, respectively (Fig. 6). Furthermore, three significant pathways were found in JH24 *vs*. JH4-1 by enrichment analysis, namely photosynthesis-antenna proteins pathway, photosynthesis pathway, and valine, leucine and isoleucine degradation pathway. In JH24 *vs*. JH4-2, in addition to annotating to these three pathways, four KEGGs of thiamine metabolism pathway, inositol phosphate metabolism pathway, glycine, serine and threonine metabolism pathway, and glyoxylate and dicarboxylate metabolism pathway were annotated. Similarly, three enrichment pathways were found in TN24 *vs*. TN4-1, including photosynthesis pathway, photosynthesis-antenna proteins pathway and proteasome pathway. More enrichment pathways were found in TN24 *vs*. TN4-2, including oxidative phosphorylation pathway, ubiquitin mediated proteolysis pathway and inositol phosphate metabolism pathway when treated at 4 °C for longer duration (Fig. 7).

### Common and specific cold resistance pathways

Two common pathways, photosynthesis-antenna proteins pathway and photosynthesis pathway, were found by comparing the KEGG enrichments from different comparison groups of JH24 *vs*. JH4-1, JH24 *vs*. JH4-2, TN24 *vs*. TN4-1, and TN24 *vs*. TN4-2. Further analysis of the annotated pathways showed that they have the same up-down genes mode pattern, suggesting that these two pathways are the primary protection response pathways to cold resistance in mango (Figs. S1 and S2). However, in addition to these two common pathways, it was found that the cold-resistant JH mango was annotated to a meaningful pathway, valine, leucine and isoleucine degradation pathway when treated with low temperature for 36 h. Furthermore, more specific pathways were identified and obtained in JH when treated with low temperature for 72 h, such as thiamine metabolism pathway, inositol phosphate metabolism pathway, glycine, serine and threonine metabolism pathway, and glyoxylate and dicarboxylate metabolism pathway (Table 4), indicating that JH mango variety has the specific cold resistance regulation pathways to cold stress.

**Table 4:** The list of KEGG enrichment pathways between 4 °C treatment group and control group in different mango varieties.

Groups comparison	KEGG B class	KEGG ID	Pathway	q value	Gene number
JH24 *vs*. JH4-1	Energy metabolism	ko00196	Photosynthesis–antenna proteins	2.40E–11	22
	Energy metabolism	ko00195	Photosynthesis	8.81E–11	49
	Amino acid metabolism	ko00280	Valine, leucine and isoleucine degradation^①^	2.11E–03	51
JH24 *vs*. JH4-2	Energy metabolism	ko00195	Photosynthesis	1.51E–09	54
	Energy metabolism	ko00196	Photosynthesis–antenna proteins	2.36E–09	22
	Amino acid metabolism	ko00280	Valine, leucine and isoleucine degradation^①^	6.95E–03	59
	Metabolism of cofactors and vitamins	ko00730	Thiamine metabolism^①^	8.54E–03	29
	Carbohydrate metabolism	ko00562	Inositol phosphate metabolism^①^	9.16E–03	75
	Amino acid metabolism	ko00260	Glycine, serine and threonine metabolism^①^	1.39E–02	54
	Carbohydrate metabolism	ko00630	Glyoxylate and dicarboxylate metabolism^①^	1.55E–02	65
TN24 *vs*. TN4-1	Energy metabolism	ko00195	Photosynthesis	2.17E–05	40
	Energy metabolism	ko00196	Photosynthesis–antenna proteins	1.29E–04	16
	Folding, sorting and degradation	ko03050	Proteasome	2.93E–02	38
TN24 *vs*. TN4-2	Energy metabolism	ko00196	Photosynthesis–antenna proteins	2.72E–06	20
	Energy metabolism	ko00195	Photosynthesis	1.58E–05	47
	Folding, sorting and degradation	ko03050	Proteasome	1.16E–02	48
	Energy metabolism	ko00190	Oxidative phosphorylation	1.18E–02	85
	Folding, sorting and degradation	ko04120	Ubiquitin mediated proteolysis	1.70E–02	128
	Carbohydrate metabolism	ko00562	Inositol phosphate metabolism	3.38E–02	73

**Note:**

① Specific KEGG pathways resistance to cold stress in JH mango varieties.

### Phylogenetic relationship of ERFs in JH mango

**Figure 8:**
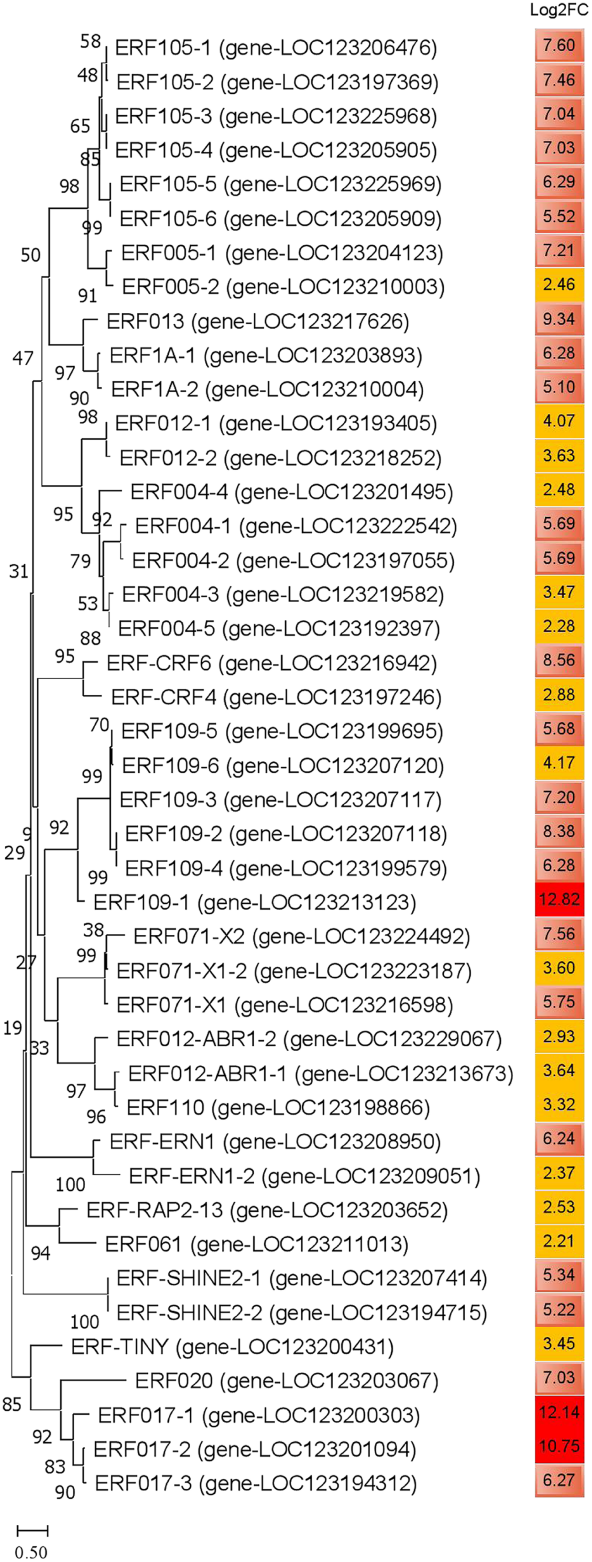
Phylogenetic analysis of amino acid sequences of ethylene-responsive transcription factors in JH mango plant. The evolutionary history was inferred by using the Maximum Likelihood method and JTT matrix-based model. The percentage of trees in which the associated taxa clustered together is shown next to the branches. This analysis involved 43 amino acid sequences. All positions with less than 75% site coverage were eliminated. There were a total of 123 positions in the final dataset. Note: dark red color represents log_2_FC ≥10; light red color represents 5.0 ≤ log_2_FC ≤ 9.9; yellow color represents 2.0 ≤ log_2_FC ≤ 4.9.

Compared with the control group, a total of 43 ERFs genes were significantly annotated into DEGs in JH mango after cold-treated for 72 h. The log_2_FC values of these DEGs were ≥2.0, and three of them ERF109-1, ERF017-1 and ERF017-2 were ≥10.0. In addition, the log_2_FC values of 24 ERFs were 5~10, and the other 16 ERFs were ≤5. The phylogenetic tree showed that the homologous genes were classify into a sub-cluster, *i.e*., ERF105-1~6, ERF109-1~6, ERF004-1~5, indicating homologous proteins have similar role or complementary function (Fig. 8). Interestingly, the up-regulated levels of ERF105-1~6 were relatively high and consistent, with log_2_FC values ranging from 5.52~7.60. Furthermore, the expression levels of ERF017-1~3 were highly up-regulated, and the log_2_FC value of ERF017-1, ERF017-2 were 12.14 and 10.75, respectively. These significantly up-regulated ERFs genes may play important regulatory roles in plant to cold resistance.

## Discussion

In the study, the key step of identification and evaluation of plant cold resistance is to obtain cold tolerant and non-cold tolerant experimental materials. On this basis, high-throughput transcriptomic sequencing was used to identified the important DEGs and signaling pathways. Before carrying out this experiment, more mango varieties were selected for cold resistance test, such as TN, JH, Guifei, Hongyu, Xiangya, Jidan varieties *etc*. Based on the pre-screening results, a cold tolerant mango variety JH and a cold sensitive mango variety TN were selected for further study. These two mango varieties were used as experimental subjects to study the DEGs and difference KEGG pathways under cold stress and to identify the specific cold resistance regulation pathways through the combination of transcriptome high-throughput sequencing and molecular biology.

Although no obvious symptoms were observation in JH and TN mango plants when treated at 4 °C for 36 and 72 h, but leaf browning, necrosis and other cold damage phenotypes were observed when transferred back in RT for 3–5 days, and leaf rot occurred when place in RT for longer duration. Interestingly, SOD and POD activities *in vivo* displayed real-time changes to varying degrees when plants were treated at 4 °C for 36 and 72 h. Inconsistent results *in vivo* and *in vitro* indicated that the physiological and biochemical indexes were responsive in real-time when mango plants treated at low temperature, but the phenotypical symptoms were delayed.

The expression of *SOD* and *POD* genes, controlled by various environmental stresses, are important protective enzymes in plants. They can reflect the changes of metabolism and stress resistance in plants at a certain period. The results of this study showed that the SOD and POD activities of TN and JH were real-time changed in different degrees when treated at low temperature. The SOD activity decreased at two time points on low temperature treatment, while the POD activity increased. Extremes of temperatures can lead to cell damage. In this regard, SOD functions as protective enzyme during changes in temperature (Saed-Moucheshi et al., 2021). Li et al. (2024) proved that SOD activity is positively correlated with plant cold resistance, while others prove that they are negatively correlated (Raza et al., 2021). In this study, the SOD activity in mango leaves displayed a downward trend after low temperature stress. We speculate that although the activity of unit enzyme increased, the overall activity of SOD in mango leaves decreased due to the influence of the expression level, after low temperature stimulation. Interestingly, SOD activity of JH mango leaves was significantly higher than that of TN mango leaves, preliminary data indicating that JH mango variety is a more cold-resistant variety under low temperature stimulation. POD is one of the key enzymes in the stress defense system in plants under stress conditions. It works synergistically with SOD and catalase (CAT) to remove excess free radicals in the body, thereby increasing plant stress resistance (Gao et al., 2024). In this study, the POD activity of the leaves from the two mango varieties showed an increasing trend under low temperature stress, and the SOD activity increment of the cold resistant mango JH was significantly greater than that of the non-cold resistant mango variety TN, indicating that POD activity was positively correlated with the cold resistance in mango.

In this study, a large amount of DEGs were obtained from JH and TN mango plants, and KEGG annotated two common pathways in response to low temperature induction, namely the photosynthesis-antenna proteins pathway and photosynthesis pathways. Further, it was found that there was no significant difference in the annotated DEGs species and up-/down-regulated genes in these two pathways, indicating that these are basic cold resistance pathways of different mango varieties in response to low temperature induction, and play an important role in the primary cold resistance pathway of plants. Similar to these results, low temperature treatment was found to activate photosynthesis-antenna proteins and photosynthesis pathways in *Rhododendron* plants (Liu et al., 2020). Photosynthesis is a crucial process in plants that converts light energy into chemical energy, enabling them to produce carbohydrates and sustain growth. However, under cold stress, photosynthesis can be severely impaired. The photosynthesis–antenna proteins pathway plays a key role in optimizing the efficiency of photosynthetic light harvesting. This allows the plant to balance energy absorption from light and prevents excess energy from causing photodamage (Dhawi, 2024). By maintaining a balanced antenna protein system, mango plants can continue to perform photosynthesis, albeit at a reduced rate, under stressful conditions.

As previous studies showed, amino acid and carbohydrate metabolic pathways play key roles in the cold stress tolerance in plants (Zhang et al., 2017a). In this study, the KEGG enrichment analyses of DEGs correlated with amino acid and carbohydrate metabolisms were significantly annotated in JH mango variety. The three essential amino acid metabolisms, such as valine, leucine and isoleucine, are abnormal in mango metabolisms under cold stress. Similar observations have also been founded in a combined metabolome and phenome analysis of plants under cold stress conditions (Hildebrandt, 2018). To date, multi-omics combination studies have identified the vital role of these metabolic pathways, such as in *Nicotiana tabacum* leaves under cold stress condition (Song et al., 2024), in tomato under salt stress condition (Zhang et al., 2017b), in soybean under salinity stress condition (Zhao et al., 2024), and in switchgrass under drought and heat stresses conditions (Ayyappan et al., 2024). Thus, these discoveries advised that plants could be related to reducing amino acid accumulation and/or a breakdown in valine, leucine and isoleucine metabolism under adverse environment.

The inositol phosphate metabolism is one of the major enriched biological pathways detected during cold stress, mainly in JH mango varity. Inositol phosphate synthase is the key enzyme of inositol synthesis, which is a central molecule required for cell metabolism and plant growth as a precursor to a large variety of compounds. As previous studies showed, inositol phosphate metabolism was induced in leaflets of M. *falcata* under cold and dehydrant stress to confer multiple resistances to abiotic stresses (Tan et al., 2013). Nevertheless, their significant roles in cold stress tolerance in different crop plants needs more investigation.

ERF is one of the largest transcription factor families in plants and play an important regulatory role in plant growth and development and abiotic stress response. The main feature of ERF is that it contains a conserved AP2 domain to bind DNA. In recent years, *ERF* genes have been identified in a variety of plants, and involve in regulating plant gene transcription to response low temperature, drought, high temperature and salt stress (Ma, Hu & Jiang, 2024). For example, the overexpression of *PtaERF194* gene in poplar improved the resistance of plants to drought stress by enhancing water use efficiency and limiting water loss (Wang et al., 2022). Rice ERF, OsERF52, as a positive modulator in response to low temperatures, directly regulated the expression of *C-Repeat Binding Factor* (*CBF*) genes, which initiates the chilling response in rice (Xu et al., 2024). *Tetrastigma hemsleyanum*, ThERF5, ThERF31, ThERF46, and ThERF55 were demonstrated a sensitive response to cold stress. Moreover, transient overexpression and RNA interference indicated that ThERF46 has a specific tolerance to cold stress (Xie et al., 2022). In addition, *Limonium bicolor* L. *ERF32* gene responsed to NaCl, PEG and ABA. The genes related to salt gland development and ion transport were significantly changed in *LbAP2*/*ERF32*-silenced lines (Jiang et al., 2024). From the above results, it can be seen that different ERF transcription factors respond to different abiotic stress processes. Therefore, it is of great significance to explore new ERF transcription factors and elucidate their regulatory mechanisms.

In addition, the researchers also found that the high level expression of some genes are closely related to the cold resistance, such as *putative calcium-binding protein CML19*, with log_2_FC of 13.52 in the study. Some studies have showed that calmodulin-like (CML) proteins are major calcium sensors that play a critical role in cold stimulus response in plants (Aleynova et al., 2023; Wu et al., 2023). It is expected to cultivate mango varieties with stronger cold resistance by regulating the expression of these genes through genetic engineering.

In summary, these studies provide an important theoretical and practical basis for improving the cold resistance and adaptability of mango plant, and help to promote the sustainable development of its industry.

## Conclusions

In the study, two cold resistant regulation pathways, the photosynthesis-antenna proteins pathway and photosynthesis pathway, were the commonly regulation pathways response to cold stress in the two mango varieties at the early stage. However, five specific cold resistant pathways, belonging to amino acid and carbohydrate metabolisms, were identified only in JH mango variety at late stage, indicating the specific regulation pathways in response to cold stress. Furthermore, 43 ethylene-responsive transcription factors (ERFs), which may play important regulatory roles in plant cold resistance, were significantly annotated in JH mango after cold-treated for 72 h, and three of them ERF109-1, ERF017-1 and ERF017-2 were highly expressed. This study provides new insights into the adaptation of mango to low temperature, and promotes the breeding of tropical plants to stress-resistant varieties.

## Supplemental Information

10.7717/peerj.18431/supp-1 Supplemental Information 1 Photosynthesis-antenna protein KEGG enrichment pathway between low temperature treatment group and control group in different mango varieties.

10.7717/peerj.18431/supp-2 Supplemental Information 2 Photosynthesis KEGG enrichment pathway between low temperature treatment group and control group in different mango varieties.

10.7717/peerj.18431/supp-3 Supplemental Information 3 The protein information of 43 ERFs.

10.7717/peerj.18431/supp-4 Supplemental Information 4 MIQE checklist.

## Additional Information and Declarations

### Data Availability

The following information was supplied regarding data availability: The 30 mango transcriptomic sequences are available at the National Genomics Data Center, Beijing Institute of Genomics, Chinese Academy of Sciences/China National Center for Bioinformation: CRA016366. https://ngdc.cncb.ac.cn/gsa/browse/CRA016366.
