# Supplementary material for: Identification of common and specific cold resistance pathways from cold tolerant and non-cold tolerant mango varieties
Source: PeerJ. 2024 Oct 30;12:e18431. doi: 10.7717/peerj.18431 (PMC11531256; doi:10.7717/peerj.18431)
Supplement: Supplemental Information 1 [file peerj-12-18431-s001.pdf]

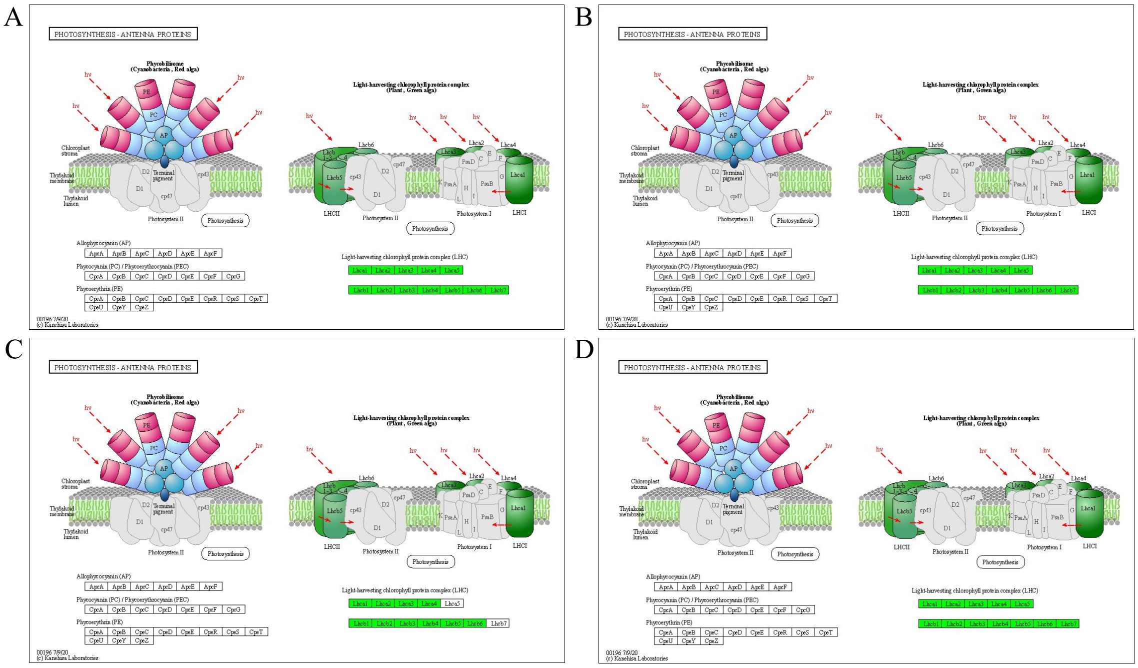

Figure S1. Photosynthesis-antenna protein KEGG enrichment pathway between low temperature treatment group and control group in different mango varieties. (A) JH24 group vs JH4-1 group, (B) JH24 group vs JH4-2 group, (C) TN24 group vs TN4-1 group, (D) TN24 group vs TN4-2 group. Note, the coloured in green represent the enzymes related to down-regulated genes.
